# Tau-Hsp70 interaction on the single molecule level

**DOI:** 10.1186/1750-1326-8-S1-P61

**Published:** 2013-10-04

**Authors:** Franziska Kundel, Magnus Kjaergaard, Sarah Shammas, Sophie Jackson, David Klenerman

**Affiliations:** 1 Department of Chemistry, University of Cambridge, Cambridge, UK

## Background

As the protein quality control centre of the cell molecular chaperones tightly regulate amyloid proteins and seem to alleviate their cytotoxicity. However, the precise mechanism of this interplay and how its modification leads to disease remain elusive.

Particularly oligomeric species of amyloid proteins have emerged as the most important mediators of neurotoxicity in neurodegenerative diseases. However, these oligomers are exceedingly hard to characterize as they are transient, heterogeneous and rare relative to the monomeric and fibrillar states. We have developed single molecule FRET techniques for the time-resolved detection and characterisation of oligomeric species [1-3].

Here, we characterise the oligomers evolving during the aggregation of a tau variant carrying the Δ280K mutation associated with some forms of frontotemporal dementia. Further, we study the interaction of these oligomers with the intracellular chaperone Hsp70 which has previously been identified as a key regulator of tau turnover.

## Materials and methods

We perform single-molecule FRET measurements for the detection of dual labelled tau oligomers. On a confocal setup two lasers of different wavelengths are overlapped to excite a sub-femtolitre confocal volume. The heparin induced tau oligomers at picomolar concentrations are flowed through the confocal volume using a microfluidic device and coincident fluorescent bursts of donor and receptor fluorophores are readily detected. Analysing the frequency and intensity of these bursts allows us to determine the amount and stoichiometries of the oligomers.

## Results

Δ280K tau oligomerisation is rapid with a maximum number of oligomers observed approximately 45 minutes after heparin induction.

In the presence of Hsp70 fewer oligomers are detected and fibrillisation is inhibited in a dose dependent manner. In more detail, more medium sized oligomers (6-20mers) and fewer small sized oligomers (2-5mers) are found in presence of the chaperone.

These effects were observed in absence of nucleotides and were found to be abrogated in the presence of ADP or ATP.

## Conclusions

Hsp70 interacts with Δ280K tau oligomers to slow down the rapid fibrillisation of the amyloid protein. This inhibitory effect seems to be due to preferential stabilization of medium sized oligomers and occurs in the absence of nucleotides.
